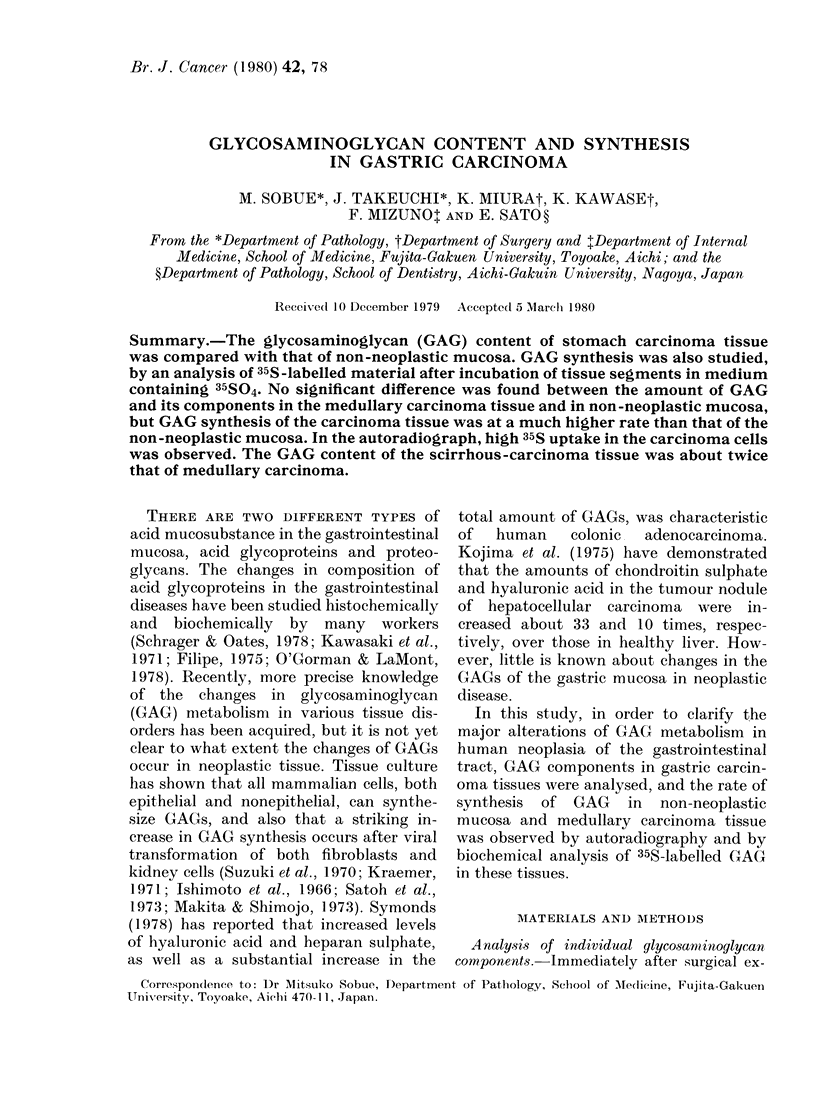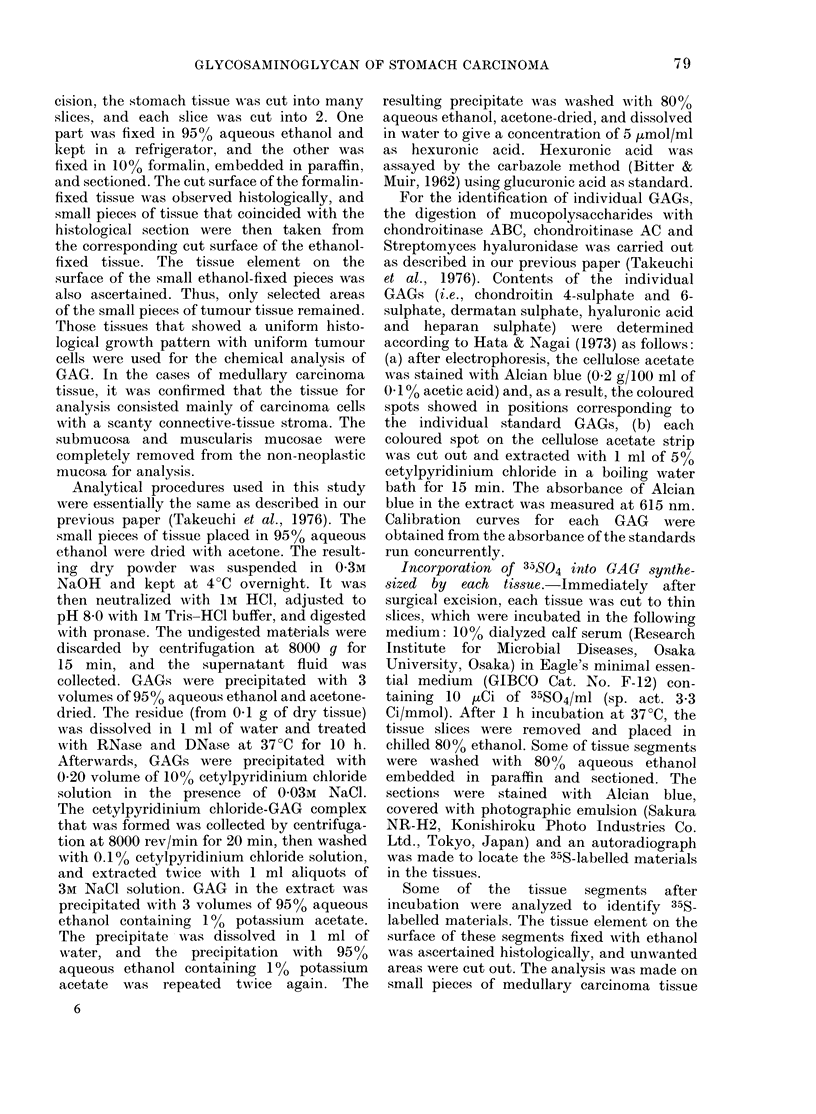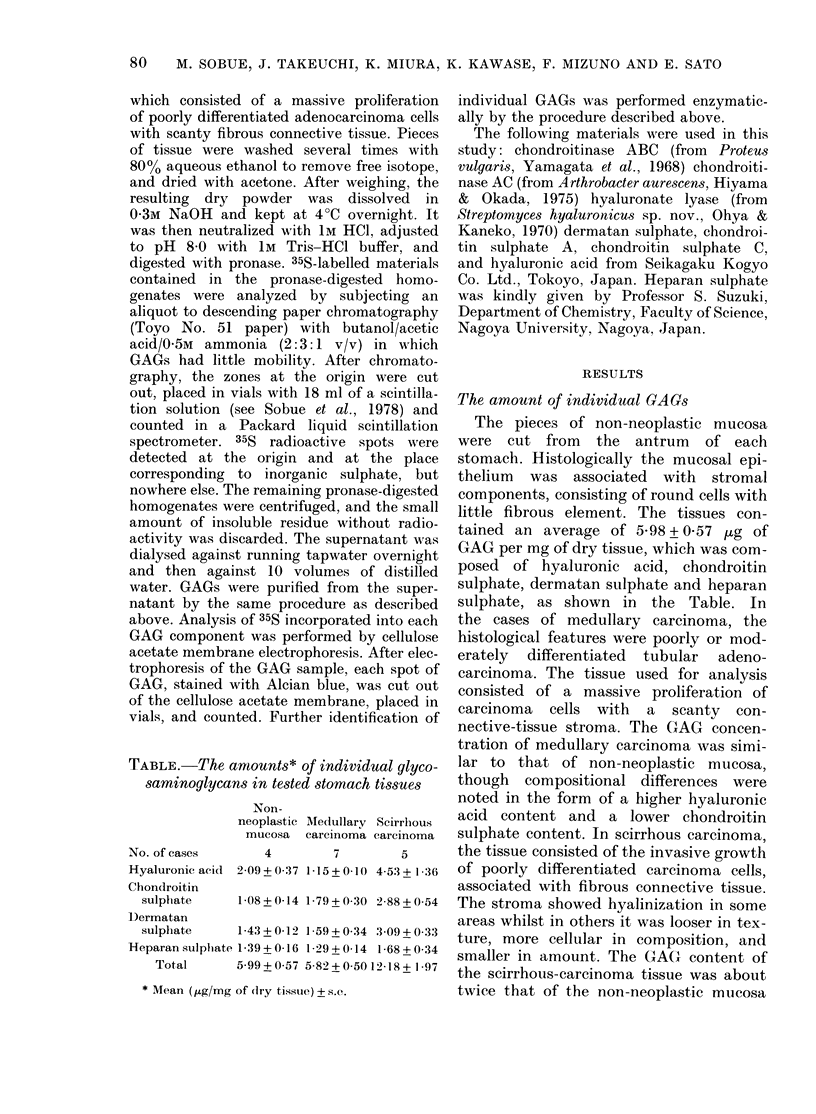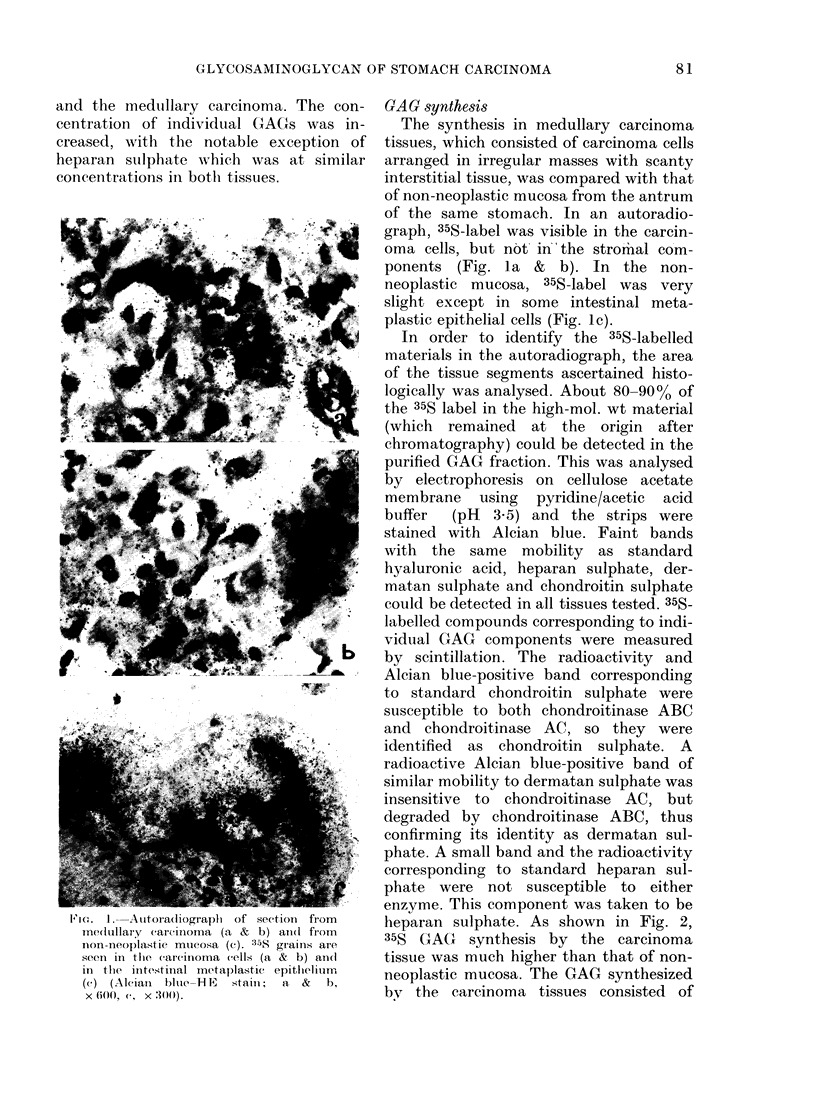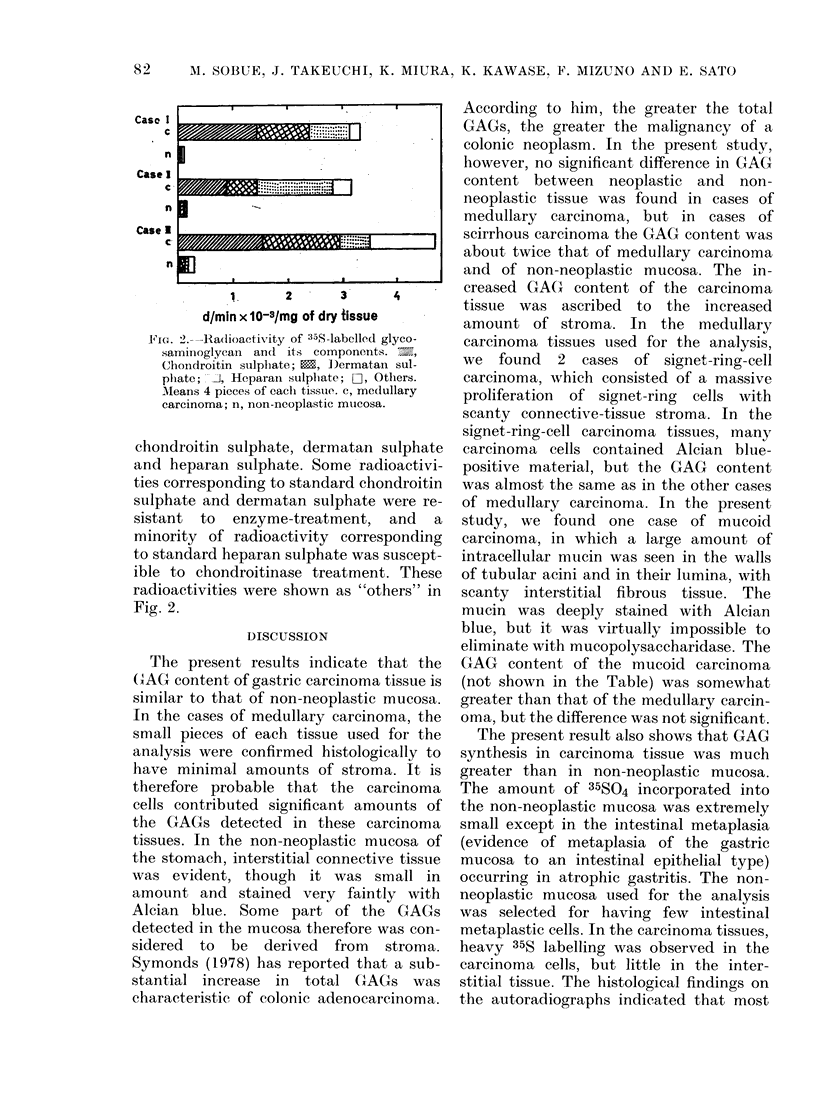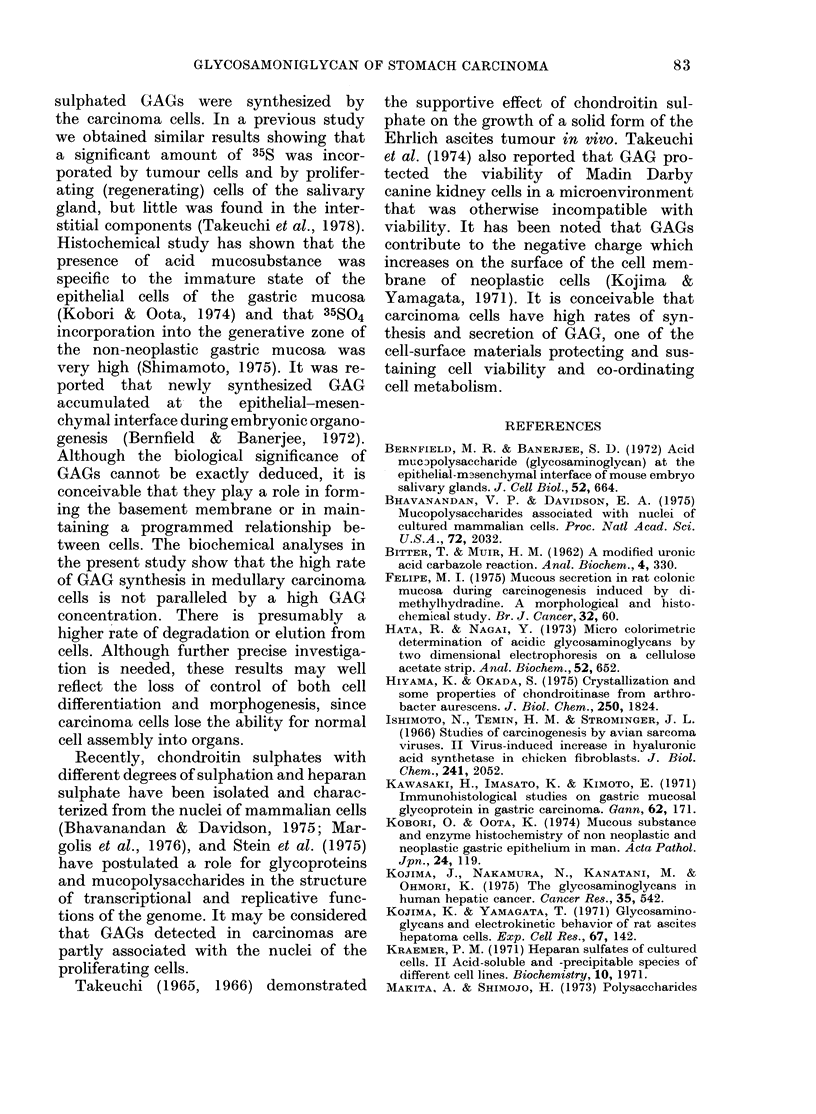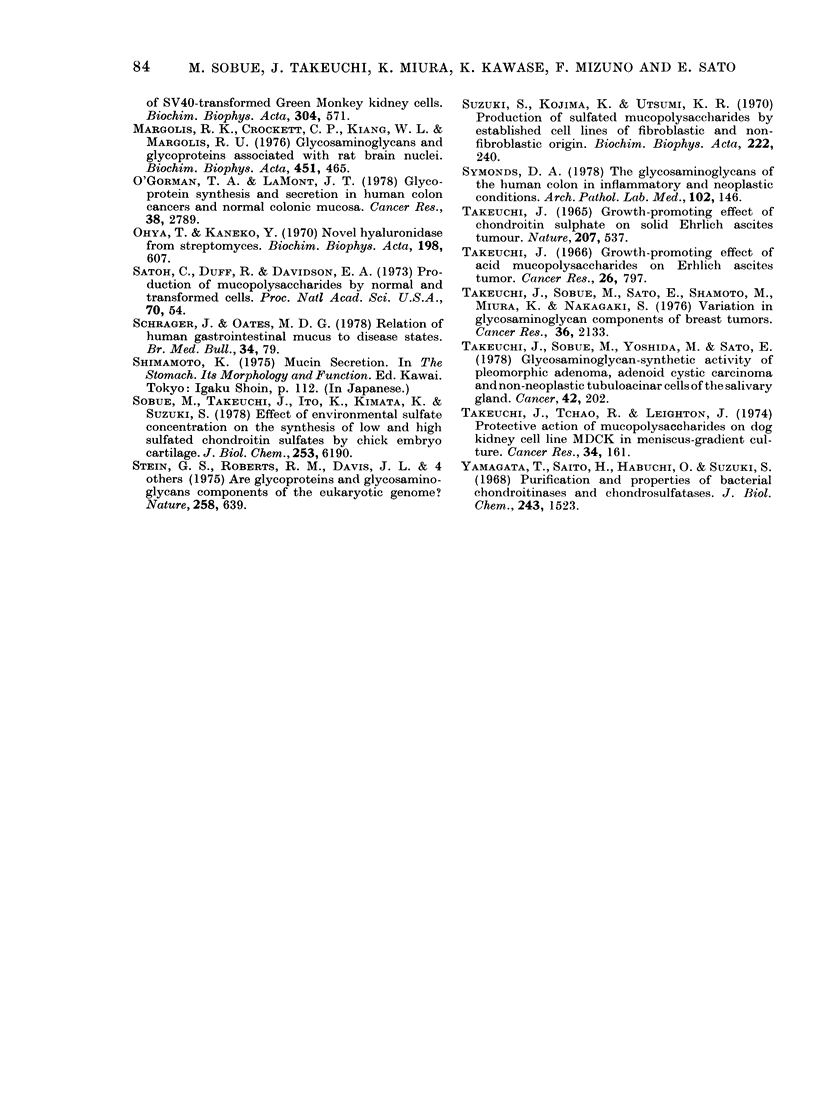# Glycosaminoglycan content and synthesis in gastric carcinoma.

**DOI:** 10.1038/bjc.1980.205

**Published:** 1980-07

**Authors:** M. Sobue, J. Takeuchi, K. Miura, K. Kawase, F. Mizuno, E. Sato

## Abstract

**Images:**


*Br. J. Cancer* (1980) **42**, 78

### GLYCOSAMINOGLYCAN CONTENT AND SYNTHESIS IN GASTRIC CARCINOMA

M. SOBUE*, J. TAKEUCHI*, K. MIURA†, K. KAWASE†,
F. MIZUNO‡ AND E. SATO§

*From the *Department of Pathology, †Department of Surgery and ‡Department of Internal Medicine, School of Medicine, Fujita-Gakuen University, Toyoake, Aichi; and the §Department of Pathology, School of Dentistry, Aichi-Gakuin University, Nagoya, Japan*

Received 10 December 1979 Accepted 5 March 1980

**Summary.—The glycosaminoglycan (GAG) content of stomach carcinoma tissue was compared with that of non-neoplastic mucosa. GAG synthesis was also studied, by an analysis of $^{35}S$-labelled material after incubation of tissue segments in medium containing $^{35}SO_4$. No significant difference was found between the amount of GAG and its components in the medullary carcinoma tissue and in non-neoplastic mucosa, but GAG synthesis of the carcinoma tissue was at a much higher rate than that of the non-neoplastic mucosa. In the autoradiograph, high $^{35}S$ uptake in the carcinoma cells was observed. The GAG content of the scirrhous-carcinoma tissue was about twice that of medullary carcinoma.**

THERE ARE TWO DIFFERENT TYPES of acid mucosubstance in the gastrointestinal mucosa, acid glycoproteins and proteoglycans. The changes in composition of acid glycoproteins in the gastrointestinal diseases have been studied histochemically and biochemically by many workers (Schrager & Oates, 1978; Kawasaki *et al.*, 1971; Filipe, 1975; O'Gorman & LaMont, 1978). Recently, more precise knowledge of the changes in glycosaminoglycan (GAG) metabolism in various tissue disorders has been acquired, but it is not yet clear to what extent the changes of GAGs occur in neoplastic tissue. Tissue culture has shown that all mammalian cells, both epithelial and nonepithelial, can synthesize GAGs, and also that a striking increase in GAG synthesis occurs after viral transformation of both fibroblasts and kidney cells (Suzuki *et al.*, 1970; Kraemer, 1971; Ishimoto *et al.*, 1966; Satoh *et al.*, 1973; Makita & Shimojo, 1973). Symonds (1978) has reported that increased levels of hyaluronic acid and heparan sulphate, as well as a substantial increase in the total amount of GAGs, was characteristic of human colonic adenocarcinoma. Kojima *et al.* (1975) have demonstrated that the amounts of chondroitin sulphate and hyaluronic acid in the tumour nodule of hepatocellular carcinoma were increased about 33 and 10 times, respectively, over those in healthy liver. However, little is known about changes in the GAGs of the gastric mucosa in neoplastic disease.

In this study, in order to clarify the major alterations of GAG metabolism in human neoplasia of the gastrointestinal tract, GAG components in gastric carcinoma tissues were analysed, and the rate of synthesis of GAG in non-neoplastic mucosa and medullary carcinoma tissue was observed by autoradiography and by biochemical analysis of $^{35}S$-labelled GAG in these tissues.

### MATERIALS AND METHODS

*Analysis of individual glycosaminoglycan components.*—Immediately after surgical ex-

Correspondence to: Dr Mitsuko Sobue, Department of Pathology, School of Medicine, Fujita-Gakuen University, Toyoake, Aichi 470-11, Japan.

cision, the stomach tissue was cut into many slices, and each slice was cut into 2. One part was fixed in 95% aqueous ethanol and kept in a refrigerator, and the other was fixed in 10% formalin, embedded in paraffin, and sectioned. The cut surface of the formalin-fixed tissue was observed histologically, and small pieces of tissue that coincided with the histological section were then taken from the corresponding cut surface of the ethanol-fixed tissue. The tissue element on the surface of the small ethanol-fixed pieces was also ascertained. Thus, only selected areas of the small pieces of tumour tissue remained. Those tissues that showed a uniform histological growth pattern with uniform tumour cells were used for the chemical analysis of GAG. In the cases of medullary carcinoma tissue, it was confirmed that the tissue for analysis consisted mainly of carcinoma cells with a scanty connective-tissue stroma. The submucosa and muscularis mucosae were completely removed from the non-neoplastic mucosa for analysis.

Analytical procedures used in this study were essentially the same as described in our previous paper (Takeuchi *et al.*, 1976). The small pieces of tissue placed in 95% aqueous ethanol were dried with acetone. The resulting dry powder was suspended in 0·3M NaOH and kept at 4°C overnight. It was then neutralized with 1M HCl, adjusted to pH 8·0 with 1M Tris–HCl buffer, and digested with pronase. The undigested materials were discarded by centrifugation at 8000 *g* for 15 min, and the supernatant fluid was collected. GAGs were precipitated with 3 volumes of 95% aqueous ethanol and acetone-dried. The residue (from 0·1 g of dry tissue) was dissolved in 1 ml of water and treated with RNase and DNase at 37°C for 10 h. Afterwards, GAGs were precipitated with 0·20 volume of 10% cetylpyridinium chloride solution in the presence of 0·03M NaCl. The cetylpyridinium chloride-GAG complex that was formed was collected by centrifugation at 8000 rev/min for 20 min, then washed with 0.1% cetylpyridinium chloride solution, and extracted twice with 1 ml aliquots of 3M NaCl solution. GAG in the extract was precipitated with 3 volumes of 95% aqueous ethanol containing 1% potassium acetate. The precipitate was dissolved in 1 ml of water, and the precipitation with 95% aqueous ethanol containing 1% potassium acetate was repeated twice again. The resulting precipitate was washed with 80% aqueous ethanol, acetone-dried, and dissolved in water to give a concentration of 5 μmol/ml as hexuronic acid. Hexuronic acid was assayed by the carbazole method (Bitter & Muir, 1962) using glucuronic acid as standard.

For the identification of individual GAGs, the digestion of mucopolysaccharides with chondroitinase ABC, chondroitinase AC and Streptomyces hyaluronidase was carried out as described in our previous paper (Takeuchi *et al.*, 1976). Contents of the individual GAGs (*i.e.*, chondroitin 4-sulphate and 6-sulphate, dermatan sulphate, hyaluronic acid and heparan sulphate) were determined according to Hata & Nagai (1973) as follows: (a) after electrophoresis, the cellulose acetate was stained with Alcian blue (0·2 g/100 ml of 0·1% acetic acid) and, as a result, the coloured spots showed in positions corresponding to the individual standard GAGs, (b) each coloured spot on the cellulose acetate strip was cut out and extracted with 1 ml of 5% cetylpyridinium chloride in a boiling water bath for 15 min. The absorbance of Alcian blue in the extract was measured at 615 nm. Calibration curves for each GAG were obtained from the absorbance of the standards run concurrently.

*Incorporation of $^{35}SO_4$ into GAG synthesized by each tissue.*—Immediately after surgical excision, each tissue was cut to thin slices, which were incubated in the following medium: 10% dialyzed calf serum (Research Institute for Microbial Diseases, Osaka University, Osaka) in Eagle's minimal essential medium (GIBCO Cat. No. F-12) containing 10 μCi of $^{35}SO_4$/ml (sp. act. 3·3 Ci/mmol). After 1 h incubation at 37°C, the tissue slices were removed and placed in chilled 80% ethanol. Some of tissue segments were washed with 80% aqueous ethanol embedded in paraffin and sectioned. The sections were stained with Alcian blue, covered with photographic emulsion (Sakura NR-H2, Konishiroku Photo Industries Co. Ltd., Tokyo, Japan) and an autoradiograph was made to locate the $^{35}S$-labelled materials in the tissues.

Some of the tissue segments after incubation were analyzed to identify $^{35}S$-labelled materials. The tissue element on the surface of these segments fixed with ethanol was ascertained histologically, and unwanted areas were cut out. The analysis was made on small pieces of medullary carcinoma tissue which consisted of a massive proliferation of poorly differentiated adenocarcinoma cells with scanty fibrous connective tissue. Pieces of tissue were washed several times with 80% aqueous ethanol to remove free isotope, and dried with acetone. After weighing, the resulting dry powder was dissolved in 0·3M NaOH and kept at 4°C overnight. It was then neutralized with 1M HCl, adjusted to pH 8·0 with 1M Tris–HCl buffer, and digested with pronase. $^{35}S$-labelled materials contained in the pronase-digested homogenates were analyzed by subjecting an aliquot to descending paper chromatography (Toyo No. 51 paper) with butanol/acetic acid/0·5M ammonia (2:3:1 v/v) in which GAGs had little mobility. After chromatography, the zones at the origin were cut out, placed in vials with 18 ml of a scintillation solution (see Sobue *et al.*, 1978) and counted in a Packard liquid scintillation spectrometer. $^{35}S$ radioactive spots were detected at the origin and at the place corresponding to inorganic sulphate, but nowhere else. The remaining pronase-digested homogenates were centrifuged, and the small amount of insoluble residue without radioactivity was discarded. The supernatant was dialysed against running tapwater overnight and then against 10 volumes of distilled water. GAGs were purified from the supernatant by the same procedure as described above. Analysis of $^{35}S$ incorporated into each GAG component was performed by cellulose acetate membrane electrophoresis. After electrophoresis of the GAG sample, each spot of GAG, stained with Alcian blue, was cut out of the cellulose acetate membrane, placed in vials, and counted. Further identification of individual GAGs was performed enzymatically by the procedure described above.

The following materials were used in this study: chondroitinase ABC (from *Proteus vulgaris*, Yamagata *et al.*, 1968) chondroitinase AC (from *Arthrobacter aurescens*, Hiyama & Okada, 1975) hyaluronate lyase (from *Streptomyces hyaluronicus* sp. nov., Ohya & Kaneko, 1970) dermatan sulphate, chondroitin sulphate A, chondroitin sulphate C, and hyaluronic acid from Seikagaku Kogyo Co. Ltd., Tokoyo, Japan. Heparan sulphate was kindly given by Professor S. Suzuki, Department of Chemistry, Faculty of Science, Nagoya University, Nagoya, Japan.

TABLE.—*The amounts* of individual glycosaminoglycans in tested stomach tissues*

| | Non-neoplastic mucosa | Medullary carcinoma | Scirrhous carcinoma |
|---|---|---|---|
| No. of cases | 4 | 7 | 5 |
| Hyaluronic acid | 2·09 ± 0·37 | 1·15 ± 0·10 | 4·53 ± 1·36 |
| Chondroitin sulphate | 1·08 ± 0·14 | 1·79 ± 0·30 | 2·88 ± 0·54 |
| Dermatan sulphate | 1·43 ± 0·12 | 1·59 ± 0·34 | 3·09 ± 0·33 |
| Heparan sulphate | 1·39 ± 0·16 | 1·29 ± 0·14 | 1·68 ± 0·34 |
| Total | 5·99 ± 0·57 | 5·82 ± 0·50 | 12·18 ± 1·97 |

* Mean (μg/mg of dry tissue) ± S.E.

### RESULTS

### *The amount of individual GAGs*

The pieces of non-neoplastic mucosa were cut from the antrum of each stomach. Histologically the mucosal epithelium was associated with stromal components, consisting of round cells with little fibrous element. The tissues contained an average of 5·98 ± 0·57 μg of GAG per mg of dry tissue, which was composed of hyaluronic acid, chondroitin sulphate, dermatan sulphate and heparan sulphate, as shown in the Table. In the cases of medullary carcinoma, the histological features were poorly or moderately differentiated tubular adenocarcinoma. The tissue used for analysis consisted of a massive proliferation of carcinoma cells with a scanty connective-tissue stroma. The GAG concentration of medullary carcinoma was similar to that of non-neoplastic mucosa, though compositional differences were noted in the form of a higher hyaluronic acid content and a lower chondroitin sulphate content. In scirrhous carcinoma, the tissue consisted of the invasive growth of poorly differentiated carcinoma cells, associated with fibrous connective tissue. The stroma showed hyalinization in some areas whilst in others it was looser in texture, more cellular in composition, and smaller in amount. The GAG content of the scirrhous-carcinoma tissue was about twice that of the non-neoplastic mucosa and the medullary carcinoma. The concentration of individual GAGs was increased, with the notable exception of heparan sulphate which was at similar concentrations in both tissues.

FIG. 1.—Autoradiograph of section from medullary carcinoma (a & b) and from non-neoplastic mucosa (c). $^{35}S$ grains are seen in the carcinoma cells (a & b) and in the intestinal metaplastic epithelium (c) (Alcian blue–HE stain; a & b, ×600, c, ×300).

*GAG synthesis*

The synthesis in medullary carcinoma tissues, which consisted of carcinoma cells arranged in irregular masses with scanty interstitial tissue, was compared with that of non-neoplastic mucosa from the antrum of the same stomach. In an autoradiograph, $^{35}S$-label was visible in the carcinoma cells, but not in the stromal components (Fig. 1a & b). In the non-neoplastic mucosa, $^{35}S$-label was very slight except in some intestinal metaplastic epithelial cells (Fig. 1c).

In order to identify the $^{35}S$-labelled materials in the autoradiograph, the area of the tissue segments ascertained histologically was analysed. About 80–90% of the $^{35}S$ label in the high-mol. wt material (which remained at the origin after chromatography) could be detected in the purified GAG fraction. This was analysed by electrophoresis on cellulose acetate membrane using pyridine/acetic acid buffer (pH 3·5) and the strips were stained with Alcian blue. Faint bands with the same mobility as standard hyaluronic acid, heparan sulphate, dermatan sulphate and chondroitin sulphate could be detected in all tissues tested. $^{35}S$-labelled compounds corresponding to individual GAG components were measured by scintillation. The radioactivity and Alcian blue-positive band corresponding to standard chondroitin sulphate were susceptible to both chondroitinase ABC and chondroitinase AC, so they were identified as chondroitin sulphate. A radioactive Alcian blue-positive band of similar mobility to dermatan sulphate was insensitive to chondroitinase AC, but degraded by chondroitinase ABC, thus confirming its identity as dermatan sulphate. A small band and the radioactivity corresponding to standard heparan sulphate were not susceptible to either enzyme. This component was taken to be heparan sulphate. As shown in Fig. 2, $^{35}S$ GAG synthesis by the carcinoma tissue was much higher than that of non-neoplastic mucosa. The GAG synthesized by the carcinoma tissues consisted of

FIG. 2.—Radioactivity of $^{35}S$-labelled glycosaminoglycan and its components. ▨, Chondroitin sulphate; ▩, Dermatan sulphate; ⬚, Heparan sulphate; □, Others. Means 4 pieces of each tissue. c, medullary carcinoma; n, non-neoplastic mucosa.

chondroitin sulphate, dermatan sulphate and heparan sulphate. Some radioactivities corresponding to standard chondroitin sulphate and dermatan sulphate were resistant to enzyme-treatment, and a minority of radioactivity corresponding to standard heparan sulphate was susceptible to chondroitinase treatment. These radioactivities were shown as "others" in Fig. 2.

### DISCUSSION

The present results indicate that the GAG content of gastric carcinoma tissue is similar to that of non-neoplastic mucosa. In the cases of medullary carcinoma, the small pieces of each tissue used for the analysis were confirmed histologically to have minimal amounts of stroma. It is therefore probable that the carcinoma cells contributed significant amounts of the GAGs detected in these carcinoma tissues. In the non-neoplastic mucosa of the stomach, interstitial connective tissue was evident, though it was small in amount and stained very faintly with Alcian blue. Some part of the GAGs detected in the mucosa therefore was considered to be derived from stroma. Symonds (1978) has reported that a substantial increase in total GAGs was characteristic of colonic adenocarcinoma. According to him, the greater the total GAGs, the greater the malignancy of a colonic neoplasm. In the present study, however, no significant difference in GAG content between neoplastic and non-neoplastic tissue was found in cases of medullary carcinoma, but in cases of scirrhous carcinoma the GAG content was about twice that of medullary carcinoma and of non-neoplastic mucosa. The increased GAG content of the carcinoma tissue was ascribed to the increased amount of stroma. In the medullary carcinoma tissues used for the analysis, we found 2 cases of signet-ring-cell carcinoma, which consisted of a massive proliferation of signet-ring cells with scanty connective-tissue stroma. In the signet-ring-cell carcinoma tissues, many carcinoma cells contained Alcian blue-positive material, but the GAG content was almost the same as in the other cases of medullary carcinoma. In the present study, we found one case of mucoid carcinoma, in which a large amount of intracellular mucin was seen in the walls of tubular acini and in their lumina, with scanty interstitial fibrous tissue. The mucin was deeply stained with Alcian blue, but it was virtually impossible to eliminate with mucopolysaccharidase. The GAG content of the mucoid carcinoma (not shown in the Table) was somewhat greater than that of the medullary carcinoma, but the difference was not significant.

The present result also shows that GAG synthesis in carcinoma tissue was much greater than in non-neoplastic mucosa. The amount of $^{35}SO_4$ incorporated into the non-neoplastic mucosa was extremely small except in the intestinal metaplasia (evidence of metaplasia of the gastric mucosa to an intestinal epithelial type) occurring in atrophic gastritis. The non-neoplastic mucosa used for the analysis was selected for having few intestinal metaplastic cells. In the carcinoma tissues, heavy $^{35}S$ labelling was observed in the carcinoma cells, but little in the interstitial tissue. The histological findings on the autoradiographs indicated that most sulphated GAGs were synthesized by the carcinoma cells. In a previous study we obtained similar results showing that a significant amount of $^{35}S$ was incorporated by tumour cells and by proliferating (regenerating) cells of the salivary gland, but little was found in the interstitial components (Takeuchi *et al.*, 1978). Histochemical study has shown that the presence of acid mucosubstance was specific to the immature state of the epithelial cells of the gastric mucosa (Kobori & Oota, 1974) and that $^{35}SO_4$ incorporation into the generative zone of the non-neoplastic gastric mucosa was very high (Shimamoto, 1975). It was reported that newly synthesized GAG accumulated at the epithelial–mesenchymal interface during embryonic organogenesis (Bernfield & Banerjee, 1972). Although the biological significance of GAGs cannot be exactly deduced, it is conceivable that they play a role in forming the basement membrane or in maintaining a programmed relationship between cells. The biochemical analyses in the present study show that the high rate of GAG synthesis in medullary carcinoma cells is not paralleled by a high GAG concentration. There is presumably a higher rate of degradation or elution from cells. Although further precise investigation is needed, these results may well reflect the loss of control of both cell differentiation and morphogenesis, since carcinoma cells lose the ability for normal cell assembly into organs.

Recently, chondroitin sulphates with different degrees of sulphation and heparan sulphate have been isolated and characterized from the nuclei of mammalian cells (Bhavanandan & Davidson, 1975; Margolis *et al.*, 1976), and Stein *et al.* (1975) have postulated a role for glycoproteins and mucopolysaccharides in the structure of transcriptional and replicative functions of the genome. It may be considered that GAGs detected in carcinomas are partly associated with the nuclei of the proliferating cells.

Takeuchi (1965, 1966) demonstrated the supportive effect of chondroitin sulphate on the growth of a solid form of the Ehrlich ascites tumour *in vivo*. Takeuchi *et al.* (1974) also reported that GAG protected the viability of Madin Darby canine kidney cells in a microenvironment that was otherwise incompatible with viability. It has been noted that GAGs contribute to the negative charge which increases on the surface of the cell membrane of neoplastic cells (Kojima & Yamagata, 1971). It is conceivable that carcinoma cells have high rates of synthesis and secretion of GAG, one of the cell-surface materials protecting and sustaining cell viability and co-ordinating cell metabolism.

### REFERENCES

Bernfield, M. R. & Banerjee, S. D. (1972) Acid mucopolysaccharide (glycosaminoglycan) at the epithelial-mesenchymal interface of mouse embryo salivary glands. *J. Cell Biol.*, **52**, 664.

Bhavanandan, V. P. & Davidson, E. A. (1975) Mucopolysaccharides associated with nuclei of cultured mammalian cells. *Proc. Natl Acad. Sci. U.S.A.*, **72**, 2032.

Bitter, T. & Muir, H. M. (1962) A modified uronic acid carbazole reaction. *Anal. Biochem.*, **4**, 330.

Felipe, M. I. (1975) Mucous secretion in rat colonic mucosa during carcinogenesis induced by dimethylhydradine. A morphological and histochemical study. *Br. J. Cancer*, **32**, 60.

Hata, R. & Nagai, Y. (1973) Micro colorimetric determination of acidic glycosaminoglycans by two dimensional electrophoresis on a cellulose acetate strip. *Anal. Biochem.*, **52**, 652.

Hiyama, K. & Okada, S. (1975) Crystallization and some properties of chondroitinase from arthrobacter aurescens. *J. Biol. Chem.*, **250**, 1824.

Ishimoto, N., Temin, H. M. & Strominger, J. L. (1966) Studies of carcinogenesis by avian sarcoma viruses. II Virus-induced increase in hyaluronic acid synthetase in chicken fibroblasts. *J. Biol. Chem.*, **241**, 2052.

Kawasaki, H., Imasato, K. & Kimoto, E. (1971) Immunohistological studies on gastric mucosal glycoprotein in gastric carcinoma. *Gann*, **62**, 171.

Kobori, O. & Oota, K. (1974) Mucous substance and enzyme histochemistry of non neoplastic and neoplastic gastric epithelium in man. *Acta Pathol. Jpn.*, **24**, 119.

Kojima, J., Nakamura, N., Kanatani, M. & Ohmori, K. (1975) The glycosaminoglycans in human hepatic cancer. *Cancer Res.*, **35**, 542.

Kojima, K. & Yamagata, T. (1971) Glycosaminoglycans and electrokinetic behavior of rat ascites hepatoma cells. *Exp. Cell Res.*, **67**, 142.

Kraemer, P. M. (1971) Heparan sulfates of cultured cells. II Acid-soluble and -precipitable species of different cell lines. *Biochemistry*, **10**, 1971.

Makita, A. & Shimojo, H. (1973) Polysaccharides of SV40-transformed Green Monkey kidney cells. *Biochim. Biophys. Acta*, **304**, 571.

MARGOLIS, R. K., CROCKETT, C. P., KIANG, W. L. & MARGOLIS, R. U. (1976) Glycosaminoglycans and glycoproteins associated with rat brain nuclei. *Biochim. Biophys. Acta*, **451**, 465.

O'GORMAN, T. A. & LAMONT, J. T. (1978) Glycoprotein synthesis and secretion in human colon cancers and normal colonic mucosa. *Cancer Res.*, **38**, 2789.

OHYA, T. & KANEKO, Y. (1970) Novel hyaluronidase from streptomyces. *Biochim. Biophys. Acta*, **198**, 607.

SATOH, C., DUFF, R. & DAVIDSON, E. A. (1973) Production of mucopolysaccharides by normal and transformed cells. *Proc. Natl Acad. Sci. U.S.A.*, **70**, 54.

SCHRAGER, J. & OATES, M. D. G. (1978) Relation of human gastrointestinal mucus to disease states. *Br. Med. Bull.*, **34**, 79.

SHIMAMOTO, K. (1975) Mucin Secretion. In *The Stomach. Its Morphology and Function.* Ed. Kawai. Tokyo: Igaku Shoin, p. 112. (In Japanese.)

SOBUE, M., TAKEUCHI, J., ITO, K., KIMATA, K. & SUZUKI, S. (1978) Effect of environmental sulfate concentration on the synthesis of low and high sulfated chondroitin sulfates by chick embryo cartilage. *J. Biol. Chem.*, **253**, 6190.

STEIN, G. S., ROBERTS, R. M., DAVIS, J. L. & 4 others (1975) Are glycoproteins and glycosaminoglycans components of the eukaryotic genome? *Nature*, **258**, 639.

SUZUKI, S., KOJIMA, K. & UTSUMI, K. R. (1970) Production of sulfated mucopolysaccharides by established cell lines of fibroblastic and non-fibroblastic origin. *Biochim. Biophys. Acta*, **222**, 240.

SYMONDS, D. A. (1978) The glycosaminoglycans of the human colon in inflammatory and neoplastic conditions. *Arch. Pathol. Lab. Med.*, **102**, 146.

TAKEUCHI, J. (1965) Growth-promoting effect of chondroitin sulphate on solid Ehrlich ascites tumour. *Nature*, **207**, 537.

TAKEUCHI, J. (1966) Growth-promoting effect of acid mucopolysaccharides on Erhlich ascites tumor. *Cancer Res.*, **26**, 797.

TAKEUCHI, J., SOBUE, M., SATO, E., SHAMOTO, M., MIURA, K. & NAKAGAKI, S. (1976) Variation in glycosaminoglycan components of breast tumors. *Cancer Res.*, **36**, 2133.

TAKEUCHI, J., SOBUE, M., YOSHIDA, M. & SATO, E. (1978) Glycosaminoglycan-synthetic activity of pleomorphic adenoma, adenoid cystic carcinoma and non-neoplastic tubuloacinar cells of the salivary gland. *Cancer*, **42**, 202.

TAKEUCHI, J., TCHAO, R. & LEIGHTON, J. (1974) Protective action of mucopolysaccharides on dog kidney cell line MDCK in meniscus-gradient culture. *Cancer Res.*, **34**, 161.

YAMAGATA, T., SAITO, H., HABUCHI, O. & SUZUKI, S. (1968) Purification and properties of bacterial chondroitinases and chondrosulfatases. *J. Biol. Chem.*, **243**, 1523.